# Experimental and Theoretical Constraints on Amino Acid Formation from PAHs in Asteroidal Settings

**DOI:** 10.1021/acsearthspacechem.1c00329

**Published:** 2022-02-15

**Authors:** Claudia-Corina Giese, Inge Loes ten Kate, Martijn P. A. van den Ende, Mariette Wolthers, José C. Aponte, Eloi Camprubi, Jason P. Dworkin, Jamie E. Elsila, Suzanne Hangx, Helen E. King, Hannah L. Mclain, Oliver Plümper, Alexander G. G. M. Tielens

**Affiliations:** † Leiden Observatory, Faculty of Science, Leiden University, 2300 RA Leiden, The Netherlands; ‡ Department of Earth Sciences, Faculty of Geosciences, Utrecht University, 3584 CB Utrecht, The Netherlands; § Université Côte d’Azur, OCA, UMR Lagrange, 06000 Nice, France; ∥ Solar System Exploration Division, NASA Goddard Space Flight Center, Greenbelt, Maryland 20771, United States; ⊥ Department of Physics, The Catholic University of America, Washington D. C. 20064, United States; # Center for Research and Exploration in Space Science and Technology, NASA/GSFC, Greenbelt, Maryland 20771, United States

**Keywords:** Polycyclic Aromatic Hydrocarbons, Amino Acids, Aqueous Alteration, Carbonaceous Chondrites, Meteorites, Equilibrium Thermodynamics

## Abstract

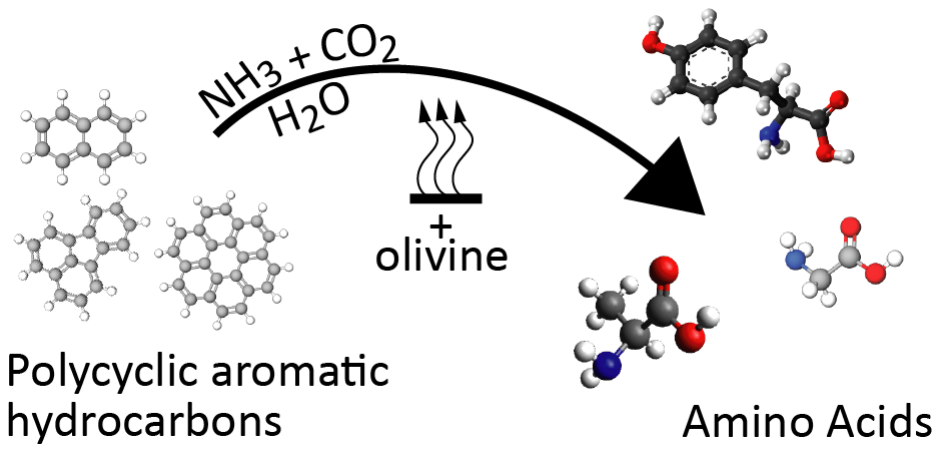

Amino acids and polycyclic aromatic hydrocarbons (PAHs) belong to the range of organic compounds detected in meteorites. In this study, we tested empirically and theoretically if PAHs are precursors for amino acids in carbonaceous chondrites, as previously suggested. We conducted experiments to synthesize amino acids from fluoranthene (PAH), with ammonium bicarbonate as a source for ammonia and carbon dioxide under mimicked asteroidal conditions. In our thermodynamic calculations, we extended our analysis to additional PAH–amino acid combinations. We explored 36 reactions involving the PAHs naphthalene, anthracene, fluoranthene, pyrene, triphenylene, and coronene and the amino acids glycine, alanine, valine, leucine, phenylalanine, and tyrosine. Our experiments do not show the formation of amino acids, whereas our theoretical results hint that PAHs could be precursors of amino acids in carbonaceous chondrites at low temperatures.

## Introduction

Carbonaceous chondrites that are found on Earth contain a multitude of organic compounds.^1−4^ Amino acids, the monomers of proteins, are well-known examples of organic molecules occurring both in terrestrial life forms and in meteorites.^5−9^ Over the years, different amino acid-forming processes in carbonaceous chondrites have been proposed and experimentally tested.^4,10−13^ The best-known reaction is the Strecker synthesis. In this synthesis, amino acids are formed through a nucleophilic addition (multistep reaction) of an aldehyde, such as formaldehyde (CH_2_O), with ammonia (NH_3_) in the presence of hydrogen cyanide (HCN) in an aqueous solution.^14−16^ An alternative yet lesser-studied theory developed by Shock and Schulte (1990)^10^ suggests that polycyclic aromatic hydrocarbons (PAHs) serve as a precursor for amino acids during hydrothermal alteration in meteorite parent bodies. These authors proposed that amino acids form during aqueous alteration reactions when considering fluoranthene or pyrene (C_16_H_10_; both PAHs) as reactants by performing calculations on the equilibrium distribution of aqueous organic compounds in metastable redox equilibria.^10^ This suggestion of the formation of amino acids from PAHs has, to our knowledge, never been tested experimentally or further investigated computationally.

Phyllosilicates have long been suggested to facilitate the alteration of organic compounds in carbonaceous chondrites.^10,17−19^ Phyllosilicates are commonly formed by serpentinization of olivine at varying temperatures.^20,21^ Over the past decades, many studies have investigated the effect of olivine or phyllosilicates on the formation or reactivity of organic compounds in various aqueous environments and temperatures.^11,22−26^ Also, in environmental sciences, olivine is used as a medium to degrade PAHs in the environment.^27,28^ However, the impact of serpentinization on PAHs under aqueous conditions is still not clear. Experiments exposing PAHs to the presence of an olivine slab at elevated temperatures have suggested a long-term chemical alteration of PAHs.^29^ Yet, a direct link between the alteration of olivine and alteration as well as reactivity of PAHs in such experiments has not been established.

To examine the feasibility of amino acid formation by PAHs and the potential catalytic role of olivine, we investigated the link between PAHs and the formation of amino acids in carbonaceous chondrites using two approaches. We performed two sets of experiments to test whether there are reaction pathways allowing for rapid amino acid formation from PAHs and whether these could be assisted by the presence of olivine under plausible asteroidal conditions at 100 and 150 °C. At the same time, we investigated the equilibrium concentration of a wide array of amino acids with PAHs to determine which PAHs are most amenable to form specific amino acids at temperatures from 25 to 150 °C. We started with the experimental exploration and applied the experimental concentrations as starting points for our thermodynamic calculations. Later, we extended our calculations to concentrations described for asteroidal settings.

## Experimental Methods

In our experimental investigation, we studied the formation of glycine (C_2_H_5_NO_2_) from fluoranthene (C_16_H_10_) as an example reaction (eq 1), with 3 fluoranthene + 26 carbon dioxide + 37 ammonia + 22 water = 37 glycine:
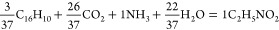
1In our theoretical investigation, we expanded our scope to other amino acid synthetic reactions.

### Experimental Study

To test the potential formation of amino acids from PAHs, we performed hydrothermal experiments on mixtures of fluoranthene, ammonium bicarbonate, and water, with and without olivine powder. For these experiments, we used polytetrafluoroethylene- (PTFE-) lined steel autoclaves (as described in Giese et al., 2019^29^) with a volume of 2 mL as reaction vessels and a standard laboratory oven (Memmert UF110, universal drying and baking oven). In total, we conducted 24 experiments, including eight blank tests. In set 1 of the experiments, we investigated the potential conversion of fluoranthene to amino acid without olivine powder, and in Set 2 the influence of a mineral surface (olivine) on this potential conversion. Set 3, consisting of two experiments with and two without olivine, was carried out to determine the pH of the mixtures. Each set of samples also contained blank tests either containing solely water (set 1) or solely water and water with olivine (set 2) (Table 1) separately placed in the oven for 206 h. We performed experiments at two temperatures, 100 °C, inspired by Shock and Schulte (1990),^10^ and 150 °C, the higher end of aqueous alteration temperatures in carbonaceous chondrites.^30^ The pressure in our experiments did not exceed 1 bar at 100 °C and 4.7 bar at 150 °C, in contrast to the 100 bar used in the model of Shock and Schulte (1990).^10^ We analyzed the 20 samples of sets 1 and 2 for their amino acid content using liquid chromatography with fluorescence detection and time-of-flight mass spectrometry (LC-FD/ToF-MS; see below for details) and the four samples of Set 3 for the pH of the samples. These experiments were performed in the same way as the two aforementioned sets. The properties of the starting concentrations and conditions are summarized in Table 1.

**Table 1:** Overview of All Experiments Performed with the Corresponding LC-FD/ToF-MS Results^a^

					AccQ-Tag results [10^–8^ M]			
no.	fluoranthene [g]	ammonium bicarbonate [g]	olivine [g]	*T* [°C]	serine	glycine	alanine	proline
set 1								
1	0.003	0.003	–	100	<0.01	<0.01	<0.01	<0.01
2	0.003	0.003		100	<0.01	<0.01	<0.01	<0.01
3	0.003	0.003		100	<0.01	<0.01	<0.01	<0.01
4	0.003	0.003		100	1.65 ± 0.14	3.11 ± 0.35	0.73 ± 0.04	0.45 ± 0.35
5				100	<0.01	<0.01	<0.01	<0.01
6	0.003	0.003		150	<0.01	<0.01	<0.01	<0.01
7	0.003	0.003		150	<0.01	<0.01	<0.01	<0.01
8	0.003	0.003		150	<0.01	<0.01	<0.01	<0.01
9				150	<0.01	<0.01	<0.01	<0.01

					AccQ-Tag Results [10^–8^ M]			
no.	fluoranthene [g]	ammonium bicarbonate [g]	olivine [g]	*T* [°C]	serine	glycine	alanine	proline
set 2								
1	0.003	0.003	0.01	100	<0.01	<0.01	<0.01	<0.01
2	0.003	0.003	0.01	100	<0.01	<0.01	<0.01	<0.01
3			0.01	100	<0.01	<0.01	<0.01	<0.01
4			0.01	100	<0.01	<0.01	0.12 ± 0.01	<0.01
5				100	–	–	–	–
6	0.003	0.003	0.01	150	<0.01	<0.01	<0.01	<0.01
7	0.003	0.003	0.01	150	<0.01	<0.01	<0.01	<0.01
8	0.003	0.003	0.01	150	<0.01	<0.01	<0.01	<0.01
9			0.01	150	<0.01	<0.01	<0.01	<0.01
10			0.01	150	<0.01	<0.01	<0.01	<0.01
11				150	<0.01	<0.01	0.17 ± 0.01	<0.01

no.	fluoranthene [g]	ammonium bicarbonate [g]	olivine [g]	*T* [°C]	pH
set 3					
1	0.003	0.003		100	9.8
2	0.003	0.003	0.01	100	10.0
3	0.003	0.003		150	9.1
4	0.003	0.003	0.01	150	9.0

a Two sets of experiments were performed, where we exposed mixtures of fluoranthene, water, and ammonium bicarbonate without (set 1) or with (Set 2) olivine powder to 100 and 150 °C. The starting material has been exposed to a temperature (*T*) of either 150 or 100 °C in anoxic (argon) atmosphere for 206 h. All experiments contained 1 mL of anoxic water. Experiment 5 of set 2 has not been analyzed due to an error in the experiment procedure. Experiments 5 and 9 of set 1 and experiments 3, 4, 9, 10, and 10 of set 2 are blank tests. The detection limit is at 10^–8^ M. Set 3 was solely performed to be used to carry out pH measurements, and were not further analyzed after the pH measurement.

#### Materials and Sample Handling

For the experiments, we used fluoranthene (C_16_H_10_; crystals, Sigma-Aldrich, 98% purity) and ammonium bicarbonate (NH_4_HCO_3_; Alfa Aesar, 99% purity) with olivine ((Mg,Fe)_2_ SiO_4_) powder of a grain size less than 125 μm (Fo90; San Carlos quarry, AZ, USA). The olivine powder was made by breaking, grinding, and sieving olivine single crystals. Subsequently, we cleaned the olivine powder with 1.2 M HCl in an ultrasonic bath. This process included the removal of the finest particles that were floating on the suspension surface. After this wet cleaning step, we baked the olivine powder in aluminum foil at 500 °C for at least 12 h. The dry baking should have no alteration effect on the olivine as it is far below the melting point of olivine but was used to remove organic compounds. In addition, no change in coloration was observed that could imply oxidation of the olivine powder (see Figure S1, picture C). We performed the experiments with anoxic water, which was obtained by bubbling 400 mL of ultrapure distilled water with argon gas using a sparger for 6 h. All steps involving fluoranthene were performed with a FFP3 mask and gloves under a fume hood, due to the carcinogenic nature of the PAH material.

First, we filled the reaction vessels with 3 mg of fluoranthene, 3 mg of ammonium bicarbonate, and 10 mg of olivine powder under the laboratory atmosphere. Then, we filled the reaction vessels with 1 mL anoxic water within an argon-filled glovebag. To obtain a headspace as oxygen-free as possible, we closed the reaction vessels in the argon atmosphere of the glovebag.

We took special care to avoid contamination in every step, from preparation and running the experiments to extraction and analysis of the sampled material. The used equipment was first cleaned with water and soap and then bathed in a two-percentage Decon90 solution for at least 12 h. Then, every piece of equipment was rinsed with ultrapure water, separately wrapped in aluminum foil, dried in the oven, subsequently autoclaved, and finally baked between 90 and 500 °C (depending on the material). The glovebag was separately cleaned and not baked. The work in the glovebag was performed with nitrile gloves, and unnecessary contact with the glovebag was avoided. We also bathed the aluminum foil before use in diluted Decon90 solution for up to 12 h and dried and baked at 500 °C in the lab oven. We took further precautions by using nitrile gloves and a FFP3 mask at every step of the procedure and by working in a clean room.

At the end of the experiments, samples were stored in Greiner centrifuge tubes sealed with aluminum foil underneath the lid. We extracted the samples and transferred them over to the Greiner tubes under argon atmosphere within a glovebag. To ensure that all material had been transferred from the experimental vessels to the centrifuge tubes, we rinsed them twice with 1–2 mL warm (below 50 °C) autoclaved, anoxic, and ultrapure water. The extracted material included the supernatant fluid as well as the solid residue. The sealed centrifuge tubes were short-term stored (few days) in a fridge at 5 °C. For the analyses, we freeze-dried the sample material under a constant nitrogen-stream at a temperature of −50 °C and pressure of 2.3–2.7 kPa. Afterward the samples were immediately shipped and analyzed for amino acids at NASA Goddard Space Flight Center (GSFC).

We performed all pH measurements at room temperature and pressure. All analyzed samples underwent the same preparation and extraction steps except for the samples whose pH was measured (set 3), which were not freeze-dried and were not further analyzed after the pH measurement.

#### Analyses of Amino Acids

Immediately after NASA GSFC received the samples, they were placed in a 4 °C refrigerator. Before the samples were further analyzed, 100 μL of ultraclean (Millipore Milli-Q Integral 10, <3 ppm total organic carbon and 18.2 MΩcm type 1 polished) water was added to each vial, which was then vortexed and centrifuged for 5 min. A portion of the supernatant (10 μL) was drawn off, and 20 μL of 0.1 M sodium borate solution was added to it; this sample was then dried under vacuum to remove any excess of volatile components. Once the sample was dry, it was brought up in 80 μL of sodium borate and 20 μL of Waters AccQ•Tag derivatizing agent.^11^ A set of eight calibrators of proteinogenic amino acid standards (0.25 to 250 μM) was also prepared similarly. Amino acids were derivatized for 10 min at 55 °C and analyzed as described in Vinogradoff et al. (2020)^11^ and Boogers et al. (2008).^31^

Once the AccQ•Tag derivatization was completed and the results obtained, samples containing greater than 1 × 10^–8^ M of amino acids were analyzed using the more laborious *o*-phthaldialdehyde/*N*-acetyl-l-cysteine (OPA/NAC) label on the same LC-FD/ToF-MS instrument as above. Amino acids were derivatized 15 min at room temperature and analyzed as described in Glavin et al. (2018).^4^ The above AccQ•Tag method, though fast, excellent for quantitation, insensitive to salts, and able to label secondary amino acids, is not able to assess chirality. The relative abundance ratio of d- and l-enantiomers of each amino acid can indicate if amino acids have formed biotically or abiotically. Biologically formed amino acids are predominantly l-enantiomers. An abundance ratio of d- and l- enantiomers skewed toward l-enantiomers would, therefore, indicate biological contamination. Conversely, an equal abundance ratio of d- and l- enantiomers would indicate an abiotic or experimental formation of amino acids. Strecker synthesis in the absence of a chiral driving force produces racemic mixtures of chiral amino acids (d/l = 1), whereas biology uses predominantly the l-isomers of amino acids; thus, contamination from terrestrial biology lowers the d/l ratio. We considered a mixture racemic if the d/l ratio is unity within 1σ (d/l approximately 0.9–1.0).

### Thermodynamic Study

Equilibrium calculations were performed to estimate the expected yield of the reactions relevant for our experiments. In our thermodynamic calculations, we extended our analysis to additional PAH–amino acid combinations. We studied 36 reactions, involving the PAHs naphthalene (C_10_H_8_), anthracene (C_14_H_10_), fluoranthene (C_16_H_10_), pyrene (C_16_H_10_), triphenylene (C_18_H_12_), and coronene (C_24_H_12_) and the amino acids glycine (C_2_H_5_NO_2_), alanine (C_3_H_7_NO_2_), valine (C_5_H_11_NO_2_), leucine (C_6_H_13_NO_2_), phenylalanine (C_9_H_11_NO_2_), and tyrosine (C_9_H_11_NO_3_). The chosen PAHs represent a selection of compounds found in carbonaceous chondrites.^32,33^ However, the chosen amino acids, glycine, alanine, valine, and leucine, are also representative of amino acids in carbonaceous chondrites,^1^ but phenylalanine and tyrosine were selected because of their aromatic structure. The physical and thermodynamic properties of these are listed in Table 2. Table 3 contains the thermodynamic data of the ammonium bicarbonate, CO_2_, NH_3_, and H_2_O species used in the calculations. For simplicity and tractability, we made the following assumptions: first, we assume strictly isobaric (constant pressure) reactions with no phase changes. Second, all aqueous and gaseous phases are assumed to behave ideally, which is valid considering the starting concentrations in this experiment (maximum ionic strength <0.04 M). Third, we treat the PAH as the main carbon source for amino acid formation, and CO_2_ as an additional carbon source or product to balance the reaction equations. Last, the only reactions considered in the present study are those listed in Table 4, though imaginably there could exist others that may affect the overall energetic favorability of the systems investigated.

**Table 2:** Physical Properties and Thermodynamic Data, Relevant for This Study, of Polycyclic Aromatic Hydrocarbons and Amino Acids at 298.15 K^a^

		Δ_*f*_*H*° [kJ/mol]	*S*° [kJ/mol]	*c*_*p*_ [kJ/molK]	melting point [°C]^34^	Boiling point [°C]^34^	solubility in water [mol/L]^34^
naphthalene	C_10_H_8_	78.53^35^	0.17^34^	0.17^34^	80.2	218	3.94 × 10^–4^
anthracene	C_14_H_10_	96.10^35^	0.25^34^	0.22^34^	216.0	314	2.04 × 10^–7^
fluoranthene	C_16_H_10_	129.20^34^	0.21^34^	0.21^34^	110.2	380	1.91 × 10^–6^
pyrene	C_16_H_10_	189.90^34^	0.23^34^	0.23^34^	150.6	394	9.23 × 10^–7^
triphenylene	C_18_H_12_	125.50^35^	0.22^36^	0.23^36^	197.8	425	2.17 × 10^–7^
coronene	C_24_H_12_	151.80^35^	0.25^36^	0.26^36^	437.3	525	3.20 × 10^–10^
glycine	C_2_H_5_NO_2_	–528.50^34^	0.28^37^	0.31^38^	290.0	decomposed^b^	7.83 × 10^–1^
alanine	C_3_H_7_NO_2_	–604.00^34^	0.10^37^	0.10^38^	297.0	decomposed^b^	5.62 × 10^–1^
valine	C_5_H_11_NO_2_	–617.90^34^	0.13^39^	0.12^38^	315.0	sublimated^b^	2.81 × 10^–1^
leucine	C_6_H_13_NO_2_	–637.40^34^	0.18^39^	0.17^38^	293.0	sublimated^b^	8.12 × 10^–2^
phenylalanine	C_9_H_11_NO_2_	–466.90^34^	0.21^34^	0.20^38^	283.0	decomposed^b^	9.86 × 10^–2^
tyrosine	C_9_H_11_NO_3_	–685.1^34^	0.21^34^	0.20^38^	343.0	decomposed^b^	1.48 × 10^–3^

a Δ_*f*_*H*° = enthalpy of formation, *S*° = standard molar entropy, and *c_p_* = heat capacity.

b PAH is either decomposed or sublimated above its melting point.

**Table 3:** Thermodynamic Data of the NH_4_HCO_3_–NH_3_–CO_2_ System at 298.15 K with Δ_*f*_*H* = Enthalpy of Formation, *S*° = Standard Molar Entropy, and *c*_*p*_ = Heat Capacity Relevant for This Study^a^

						polynomial coefficients for Shomate equations^40^							
			Δ_*f*_*H* [kJ/mol]	*S*° [kJ/mol]	*c*_*p*_ [kJ/molK]	*a*	*b*	*c*	*d*	*e*	*f*	*g*	*h*
ammonium bicarbonate	NH_4_ HCO_3_	(s)	–849.4^41^	0.12^41^									
carbon dioxide	CO_2_	(aq)	–413.26^34^	0.12^34^	0.28^34^								
carbon dioxide	CO_2_	(g)	–393.51^40^	0.21^40^		25	55.19	–33.69	7.95	–0.14	–403.61	228.24	–393.52
carbonate	CO_3_^2–^	(aq)	–677.14^41^	–0.06^41^	–0.23^41^								
bicarbonate	HCO_3_^–^	(aq)	–691.99^34^	0.09^34^	0.09^34^								
carbonic acid	H_2_CO_3_	(s)	–699.65^41^	0.19^41^									
ammonia	NH_3_	(aq)	–81.15^41^	0.11^41^									
ammonia	NH_3_	(g)	–45.9^40^	0.19^40^		20	49.77	–15.38	1.92	0.19	–53.31	203.86	–45.9
ammonium	NH_4_^+^	(aq)	–132.5^34^	0.11^34^	0.08^34^								
water	H_2_O	(l)	–285.83^40^	0.07^40^		–203.61	1523.29	–3196.41	2474.46	3.86	–256.55	–488.72	–285.83
hydroxide ion	OH^–^	(aq)	–229.994^41^	–0.01^41^									
hydrogen	H^+^	(aq)	0^41^	0^41^									

a The polynomial coefficients for the Shomate equations were used for the calculation of the enthalpy and entropy (eq 4 and 5) of CO_2_, NH_3_, and H_2_O.

**Table 4:** Gibbs Free Energy of Reaction Δ*G*_*R*_ [kJ/mol] and Equilibrium Constants log *K*_*c*_ for the Formation of 1 mol of Amino Acid at 25, 100, and 150 °C^a^

	Δ*G*_*R*__,25_	Δ*G*_*R*__,100_	Δ*G*_*R*__,150_	log *K*_*c*__,25_	log *K*_*c*__,100_	log *K*_*c*__,150_
naphthalene						
^1^/_8_C_1__0_H_8_ + ^3^/_4_CO_2_ + 1NH_3_ + ^1^/_2_H_2_O = 1C_2_H_5_NO_2_	40.5	53.5	63.6	–7.1	–7.5	–7.9
^1^/_4_C_1__0_H_8_ + ^1^/_2_CO_2_ + 1NH_3_ + 1H_2_O = 1C_3_H_7_NO_2_	–5.2	7.4	17.0	0.9	–1.0	–2.1
^1^/_2_C_1__0_H_8_ + 1NH_3_ + ^1^/_2_H_2_O = 1C_5_H_1__1_NO_2_	40.9	53.0	61.7	–7.2	–7.4	–7.6
^5^/_8_C_1__0_H_8_ + 1NH_3_ + 1^5^/_2_H_2_O = 1C_6_H_1__3_NO_2_ + ^1^/_4_CO_2_	49.1	60.2	67.8	–8.6	–8.4	–8.4
^5^/_6_C_1__0_H_8_ + ^2^/_3_CO_2_ + 1NH_3_ + ^2^/_3_H_2_O = 1C_9_H_1__1_NO_2_	63.0	76.7	87.3	–11.0	–10.7	–10.8
^1^^9^/_2__4_C_1__0_H_8_ + ^1^^1^/_1__2_CO_2_ + 1NH_3_ + ^5^/_6_H_2_O = 1C_9_H_1__1_NO_3_	83.9	102.7	117.7	–14.7	–14.4	–14.5
anthracene						
^1^/_1__1_C_1__4_H_1__0_ + ^8^/_1__1_CO_2_ + 1NH_3_ + ^6^/_1__1_H_2_O = 1C_2_H_5_NO_2_	41.5	55.3	66.0	–7.3	–7.6	–7.9
^2^/_1__1_C_1__4_H_1__0_ + ^5^/_1__1_CO_2_ + 1NH_3_ + 1^1^/_1__1_H_2_O = 1C_3_H_7_NO_2_	–2.9	11.1	21.9	0.5	–1.3	–2.3
^4^/_1__1_C_1__4_H_1__0_ + 1NH_3_ + 2^2^/_1__1_H_2_O = 1C_5_H_1__1_NO_2_ + ^1^/_1__1_CO_2_	45.1	60.4	71.6	–7.9	–7.9	–8.0
^5^/_1__1_C_1__4_H_1__0_ + 1NH_3_ + 2^8^/_1__1_H_2_O = 1C_6_H_1__3_NO_2_ + ^4^/_1__1_CO_2_	55.6	69.4	80.2	–9.7	–9.2	–9.0
^2^^0^/_3__3_C_1__4_H_1__0_ + ^1^^7^/_3__3_CO_2_ + 1NH_3_ + ^2^^2^/_3__3_H_2_O = 1C_9_H_1__1_NO_2_	70.6	89.0	103.8	–12.4	–11.7	–11.5
^19^/_33_C_14_H_10_ + ^31^/_33_CO_2_ + 1NH_3_ + 1^4^/_33_H_2_O = 1C_9_H_11_NO_3_	90.9	112.8	131.7	–15.9	–15.2	–15.1
fluoranthene						
^3^/_3__7_C_1__6_H_1__0_ + ^2^^6^/_3__7_CO_2_ + 1NH_3_ + ^2^^2^/_3__7_H_2_O = 1C_2_H_5_NO_2_	42.3	55.1	65.0	–7.4	–7.7	–7.9
^6^/_3__7_C_1__6_H_1__0_ + ^1^^5^/_3__7_CO_2_ + 1NH_3_ + ^4^/_3__7_H_2_O = 1C_3_H_7_NO_2_	–2.1	10.2	19.4	0.4	–1.4	–1.9
^1^^2^/_3__7_C_1__6_H_1__0_ + 1NH_3_ + 1^1^^4^/_3__7_H_2_O = 1C_5_H_1__1_NO_2_ + ^7^/_3__7_CO_2_	47.6	59.0	66.9	–8.3	–8.3	–8.3
^1^^5^/_3__7_C_1__6_H_1__0_ + 1NH_3_ + 2^3^^6^/_3__7_H_2_O = 1C_6_H_1__3_NO_2_ + ^1^^8^/_3__7_CO_2_	56.0	66.2	72.9	–9.8	–9.3	–9.1
^2^^0^/_3__7_C_1__6_H_1__0_ + ^1^^3^/_3__7_CO_2_ + 1NH_3_ + 1^1^^1^/_3__7_H_2_O = 1C_9_H_1__1_NO_2_	73.4	86.0	95.4	–12.9	–12.0	–11.9
^1^^9^/_3__7_C_1__6_H_1__0_ + ^2^^9^/_3__7_CO_2_ + 1NH_3_ + 1^1^^6^/_3__7_H_2_O = 1C_9_H_1__1_NO_3_	92.8	110.5	124.3	–16.3	–15.5	–15.4
pyrene						
^3^/_3__7_C_1__6_H_1__0_ + ^2^^6^/_3__7_CO_2_ + 1NH_3_ + ^2^^2^/_3__7_H_2_O = 1C_2_H_5_NO_2_	47.4	60.1	70.0	–8.3	–8.4	–8.6
^6^/_3__7_C_1__6_H_1__0_ + ^1^^5^/_3__7_CO_2_ + 1NH_3_ + ^4^/_3__7_H_2_O = 1C_3_H_7_NO_2_	8.1	20.3	29.4	–1.4	–2.8	–3.6
^1^^2^/_3__7_C_1__6_H_1__0_ + 1NH_3_ + 1^1^^4^/_3__7_H_2_O = 1C_5_H_1__1_NO_2_ + ^7^/_3__7_CO_2_	67.9	79.2	87.0	–11.9	–11.1	–10.7
^1^^5^/_3__7_C_1__6_H_1__0_ + 1NH_3_ + 2^3^^6^/_3__7_H_2_O = 1C_6_H_1__3_NO_2_ + ^1^^8^/_3__7_CO_2_	81.4	91.4	98.0	–14.3	–12.8	–12.1
^2^^0^/_3__7_C_1__6_H_1__0_ + ^1^^3^/_3__7_CO_2_ + 1NH_3_ + 1^1^^1^/_3__7_H_2_O = 1C_9_H_1__1_NO_2_	107.3	119.7	128.9	–18.8	–16.8	–15.9
^1^^9^/_3__7_C_1__6_H_1__0_ + ^2^^9^/_3__7_CO_2_ + 1NH_3_ + 1^1^^6^/_3__7_H_2_O = 1C_9_H_1__1_NO_3_	125.0	142.5	156.1	–21.9	–20.0	–19.3
triphenylene						
^1^/_1__4_C_1__8_H_1__2_ + ^5^/_7_CO_2_ + 1NH_3_ + ^2^/_7_H_2_O = 1C_2_H_5_NO_2_	44.2	56.9	66.8	–7.7	–8.0	–8.2
^1^/_7_C_1__8_H_1__2_ + ^3^/_7_CO_2_ + 1NH_3_ + 1^1^/_7_H_2_O = 1C_3_H_7_NO_2_	1.9	14.1	23.2	–0.3	–2.0	–2.9
^2^/_7_C_1__8_H_1__2_ + 1NH_3_ + 2^2^/_7_H_2_O = 1C_5_H_1__1_NO_2_ + ^1^/_7_CO2	57.8	69.0	76.8	–10.1	–9.7	–9.5
^5^/_1__4_C_1__8_H_1__2_ + 1NH_3_ + 2^6^/_7_H_2_O = 1C_6_H_1__3_NO_2_ + ^3^/_7_CO_2_	69.3	79.3	85.8	–12.1	–11.1	–10.6
^1^^0^/_2__1_C_1__8_H_1__2_ + ^3^/_7_CO2 + 1NH_3_ + 1^1^/_7_H_2_O = 1C_9_H_1__1_NO_2_	88.2	100.3	109.4	–15.4	–14.0	–13.5
^1^^9^/_4__2_C_1__8_H_1__2_ + ^1^^2^/_1__3_CO_2_ + 1NH_3_ + 1^2^/_7_H_2_O = 1C_9_H_1__1_NO_3_	110.0	127.3	140.9	–19.3	–17.8	–17.4
coronene						
^1^/_1__8_C_2__4_H_1__2_ + ^2^/_3_CO_2_ + 1NH_3_ + ^2^/_3_H_2_O = 1C_2_H_5_NO_2_	54.6	67.1	76.8	–9.6	–9.4	–9.5
^1^/_9_C_2__4_H_1__2_ + ^1^/_3_CO_2_ + 1NH3 + 1^1^/_3_H_2_O = 1C_3_H_7_NO_2_	21.2	32.9	41.7	–3.7	–4.6	–5.2
^2^/_9_C_2__4_H_1__2_ + 1NH_3_ + 2^2^/_3_H_2_O = 1C_5_H_1__1_NO_2_ + ^1^/_3_CO_2_	97.2	107.6	114.7	–17.0	–15.1	–14.2
^5^/_1__8_C_2__4_H_1__2_ + 1NH_3_ + 1^2^/_3_H_2_O = 1C_6_H_1__3_NO_2_ + ^2^/_3_CO_2_	116.7	125.7	131.4	–20.4	–17.6	–16.2
^1^^0^/_2__7_C_2__4_H_1__2_ + ^1^/_3_CO_2_ + 1NH_3_ + ^2^/_3_H_2_O = 1C_9_H_1__1_NO_2_	155.1	166.1	174.1	–27.2	–23.3	–21.5
^19^/_5__4_C_2__4_H_1__2_ + ^1^^5^/_2__7_CO_2_ + 1NH_3_ + 1^2^^4^/_2__7_H_2_O = 1C_9_H_1__1_NO_3_	171.6	187.8	200.3	–30.1	–26.3	–24.7

a Amino acids: glycine (C_2_H_5_NO_2_), alanine (C_3_H_7_NO_2_), valine (C_5_H_11_NO_2_), leucine (C_6_H_13_NO_2_), phenylalanine (C_9_H_11_NO_2_), and tyrosine (C_9_H_11_NO_3_). PAHs: naphthalene (C_8_H_10_), anthracene (C_14_H_10_), fluoranthene (C_16_H_10_), pyrene (C_16_H_10_), triphenylene (C_18_H_12_), and coronene (C_24_H_12_).

In the calculations, we explicitly specified if a species is in the solid (s), aqueous (aq), or gaseous (g) phase. The initial concentrations of CO_2_ and NH_3_ (which are reactants in the amino acid synthesis reactions) are derived from the initial ammonium bicarbonate concentration used in the experiment (3 mg in 1 mL = 3.79 × 10^2^ M). To not disturb and potentially contaminate our experimental mixtures, the initial pH values were not directly measured in our experiments and instead calculated with the model detailed below.

#### Temperature Correction of Enthalpy and Entropy

To obtain Gibbs free energy values for each reaction (and corresponding equilibrium constants), we need to estimate the enthalpy *H* and entropy *S* for each species at the specific temperatures relevant for our experiments. This is done either through a first-order Taylor expansion using the heat capacity *c*_*p*_ of the respective species, or with the Shomate equation assuming a constant heat capacity, applicable for fluids and gases.^40,42^ The temperature-correction relations for the enthalpy and entropy of the solutes are given by
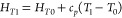
2
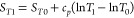
3where *c*_*p*_ is the heat capacity at constant pressure, *H*_*T*0_ and *S*_*T*0_ the standard state enthalpy and entropy at *T*_0_ = 298.15 K, *H*_*T*1_ and *S*_*T*1_ the enthalpy and entropy at the final temperature *T*_1_*.* The final temperature equals the specific temperature of interest.

The enthalpy and entropy for most gases and liquid water can be calculated with the Shomate equation (eqs 4 and 5). The polynomial coefficients are empirically determined for each species^40^ (see Table 3).
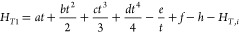
4
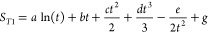
5where *a*–*h* are the specific polynomial coefficients for each species and *t* denotes the absolute temperature of interest divided by 1000. We used the standard state values in the case that neither *c*_*p*_ nor the polynomial coefficients for the Shomate equation were known.

#### Gibbs Free Energy of Reaction

Following standard definitions, we calculate the Gibbs free energy of formation *G*_*f*_ of each molecule at a given temperature *T*_*x*_ using eq 6:
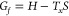
6The Gibbs free energy of reaction Δ*G*_*R*_ is the sum of the Gibbs free energy of formation *G*_*f*_ of each involved species in a reaction at a specific temperature. Δ*G*_*R*_ is calculated using eq 7:
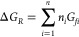
7where the summation is performed over all the species in each given reaction, with *n*_*i*_ being the stoichiometric coefficient for the *i*th species (defined positive for reaction products and negative for reactants), and *G*_*Ri*_ being its Gibbs free energy of reaction calculated using eq 6.

#### Equilibrium Constant and Theoretical Amino Acid Concentrations

After obtaining the Gibbs free energy of a reaction at a given temperature, the equilibrium constant *K*, which describes the ratio between reaction products and reactants at equilibrium, is given by the relation
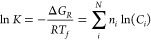
8where *R* is the gas constant, *n*_*i*_ is the stoichiometric coefficient of the *i*th species, and *C*_*i*_ is its equilibrium concentration. For the reaction forming glycine from fluoranthene (eq 1), considering an infinite diluted solution, assuming a closed system and a water activity of 1, the equilibrium condition can be specifically written as
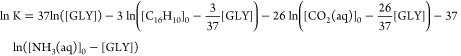
9Usually, such an equation does not contain the initial concentrations but relates the equilibrium concentrations of glycine to the equilibrium concentrations of the reactants. In eq 9, [X]_0_ denotes the initial concentration of species X, and [GLY = glycine] denotes the equilibrium concentration of glycine, assuming that all decrease in initial reactant concentration is due to production of glycine. To calculate the reactants, we subtract the reactant’s equilibrium concentration from the original concentration. This nonlinear equation (eq 9) can be solved for [GLY] using a standard root-finding algorithm. However, if the equilibrium glycine concentration is extremely small compared to the initial reactant concentrations, standard root solvers can become unstable due to finite-precision arithmetic. Fortunately, in the case of a vanishingly small glycine concentration, eq 9 can be accurately approximated by eq 10, which can be solved analytically.
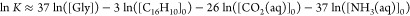
10Combining eq 10 and our calculated Δ*G*_R_ values, we can approximate the yield of the glycine synthesis reaction at equilibrium. Other amino acids were calculated using equivalent approximations. In total, 36 reactions with six amino acids (glycine, alanine, valine, leucine, phenylalanine, and tyrosine) and six PAHs (naphthalene, anthracene, fluoranthene, pyrene, triphenylene, and coronene) were calculated (Table S1). eq 1 represents our example reaction with CO_2_(aq) and NH_3_(aq), which is also used as an example by Shock and Schulte (1990).^10^ We used the values for solubility in water (Table 1) for the concentration of the PAHs. The calculated equilibrium constants are listed in Table 4.

#### CO_2_–NH_3_–H_2_O System

In our experiments, we used dissolved ammonium bicarbonate as a source for CO_2_ and NH_3_^43^ and as the starting reaction for amino acid formation. Dissolution of ammonium bicarbonate, which results in a CO_2_–NH_3_–H_2_O system, is a well-studied reaction system.^44−48^ Intermediate reactions within this system could lock up significant amounts of our starting reactants (CO_2_ and NH_3_), making them unavailable for the formation of amino acids. Such reaction intermediates, including formation of the following dissolved and gaseous species NH_3_(aq,g)/NH_4_^+^(aq), CO_2_(aq,g)/HCO_3_^–^(aq)/CO_3_^2–^(aq), and H^+^(aq)/OH^–^(aq)/H_2_O, are listed in Table S2. In addition, it can be noted that CO_2_ and NH_3_ alone do not form amino acid like structures in water (e.g., Sutter and Mazzotti (2017)^43^) without an additional energy source as tested by Harada and Suzuki (1977),^49^ for example.

The calculated H^+^(aq) concentrations were used to calculate the pH at each temperature. For all calculations, we assumed ideal behavior, where, for instance, the H^+^(aq) activity is approximated by the H^+^(aq) concentrations. To assess the effect of intermediate aqueous reactions on the formation of amino acids, we calculated the equilibrium state (solving all reactions as a function of temperature simultaneously until an equilibrium was reached) of the CO_2_–NH_3_–H_2_O system (excluding the amino acid-forming reaction) at the temperatures of interest using ChemPy.^50^ The reactions and corresponding calculated Δ*G*_R_ values are listed in Table S2.

#### A Priori Consideration

In order to form amino acids, it is apparent that a reduced nitrogen compound, such as NH_3_, and a source for the functional groups −COOH and −CH are required. We had no information on putative reaction pathways that could drive amino acid formation from PAHs, nor on their respective kinetics and limiting factors. In our theoretical approach, we treated CO_2_ optionally as additional carbon source and focused on the PAH as main provider of carbon. We decided to experimentally study this system following Shock and Schulte (1990)^10^ theoretical predictions. Shock and Schulte (1990),^10^ proposed that PAHs provide the base hydrocarbon structure (−CH) structure with which CO_2_ and NH_3_ in H_2_O can react to form amino acids. NH_3_ and CO_2_ are provided by the dissolution of ammonium bicarbonate. In our experiments, fluoranthene was added in an oversaturated amount (Table 1). Additionally, we deliberately chose water-saturated conditions to reduce the possibility of having insufficient amounts of water for any water-dependent reactivity.

## Results

### Relative Abundance of Amino Acids

We probed our sample materials for the presence of amino acids using the LC-FD/ToF-MS AccQ•Tag method. Table 1 shows that only set 1–sample 4, set 2–sample 4, and set 2–sample 1 contained small amounts of amino acids. In set 1–sample 4 we detected alanine, glycine, serine, and proline. Set 2–sample 4 and set 2–sample 11 contained a small amount of alanine. Considering set 2–sample 4 and set 2–sample 11 are blanks and did not contain fluoranthene or ammonium bicarbonate, these data suggest that the trace amounts of alanine detected here are a consequence of contamination. We determined the relative abundance of d- and l-enantiomers for set 1–sample 4 to test if the detected amino acids were a result of contamination. The measured d/l ratios were 0.82 ± 0.15 for serine, 0.53 ± 0.14 for alanine, 0.86 ± 0.33 for aspartic acid, and 0.27 ± 0.30 for glutamic acid. These abundances, particularly those of alanine and glutamic acid, indicate that the amino acids detected in set 1–sample 4 are contaminants. Furthermore, none of our amino acid measurements found detectable concentrations of amino acids suggesting other than a contamination origin.

### Experimental Observations and Measured pH

Before the experiments, the settled mixtures of fluoranthene and ammonium bicarbonate, combined with or without olivine powder, appeared white in water. At the end of the 100 °C experiments, after the transfer of the sample material from the reaction vessels, a residue was visible at the bottom of the centrifuge tubes. This residue was, however, not present in the blank tests containing only water. The extracted fluids from 150 °C experiments appeared cloudy with a white residue or yellow-brownish coloration with a white and colored layer (Figure S1). The pH of the fluids extracted after reactions of fluoranthene with ammonium bicarbonate with or without olivine varied from around 10 to around 9 (Table 1).

### CO_2_–NH_3_–H_2_O System and Calculated pH

**Figure 1:**
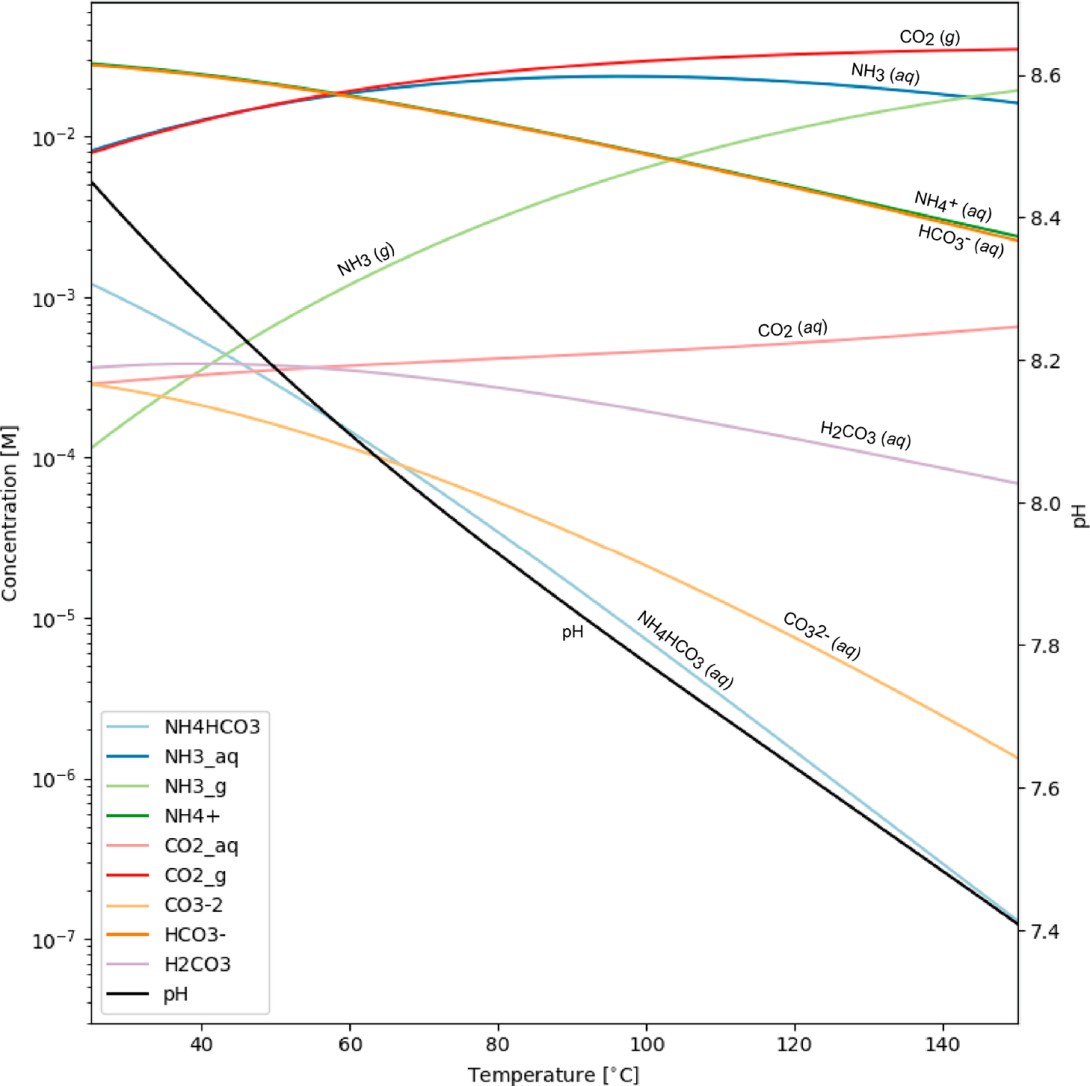
Calculated CO_2_–NH_3_–H_2_O system for the initial concentration of ammonium bicarbonate of 3.79 × 10^–2^ M at 20 °C. With subsequent increasing temperature, pH and dissolved gas concentrations evolve.

Figure 1 shows the calculated results of the CO_2_–NH_3_–H_2_O system and the corresponding pH. Within the temperature range of 25–150 °C, CO_2_ in the aqueous and gas phase, and NH_3_ in the gas phase increase with increasing temperature. In contrast, NH_3_ in the aqueous phase first increases and around 100 °C starts to decrease. At low temperature, NH_4_^+^(aq) and HCO^3–^(aq) are the dominant species but as the temperature increases, the ammonium equilibrium shifts toward NH_3_ while more NH_3_ and CO_2_ go into the gas phase. The calculated pH for the ammonium bicarbonate solutions diminished from 8.45 at 25 °C, over 7.75 at 100 °C to 7.4 at 150 °C. The concentrations of NH_3_(aq,g)/NH_4_^+^(aq), CO_2_(aq,g)/HCO_3_^–^(aq)/CO_3_^2–^(aq), and H^+^(aq)/OH^–^(aq) at 25, 100, and 150 °C, are listed in Table S3. We used these speciation calculations to determine the initial reactant concentrations (e.g., eq 9).

### Thermodynamic System and Theoretical Amino Acid Concentrations

**Figure 2:**
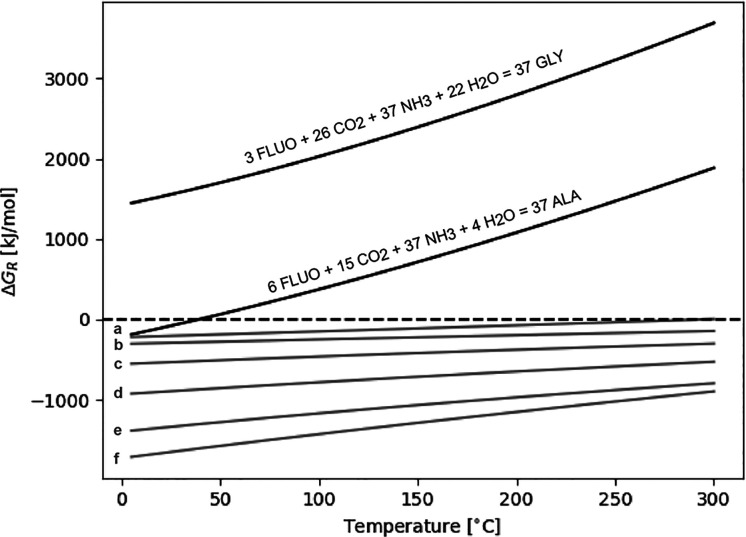
Gibbs free energy of reaction Δ*G*_*R*_ as a function of temperature for the reaction forming glycine (GLY) and alanine (ALA) formation by fluoranthene (FLUO). The gray lines represent amino acid formation (Strecker synthesis) with formaldehyde. (a) glycine formation; (b) alanine formation, (c) valine formation, (d) leucine formation, (e) phenylalanine formation, and (f) tyrosine formation (see reactions and Δ*G*_*R*_ values in Table S4). The black dashed line indicates the transition to an exergonic reaction (Δ*G*_R_ < 0).

**Figure 3:**
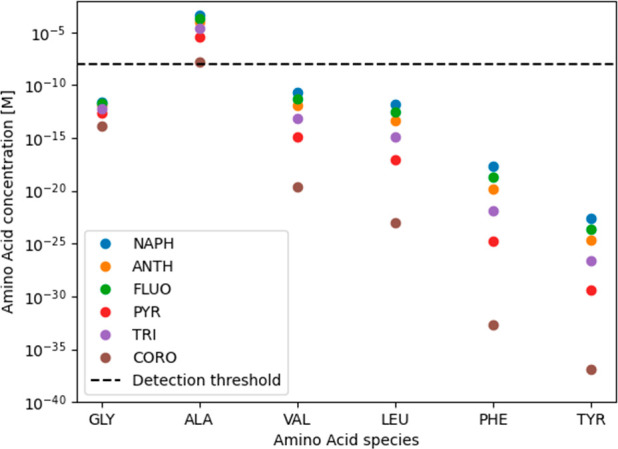
Calculated amino acid concentrations per species for all 36 reactions (Table 4) at 150 °C, sorted by amino acid molecule size. The observed trends are similar from 25 to 150 °C. Only reactions leading to the formation of alanine result in a concentration above the analytical detection limit of 1 × 10^–8^ M (dashed line). NAPH = naphthalene, ANTH = anthracene, FLUO = fluoranthene, PYR = pyrene, TRI = triphenylene, CORO = coronene; GLY = glycine, ALA = alanine, VAL = valine, LEU = leucine, PHE = phenylalanine, TYR = tyrosine

We calculated the Gibbs free energy and corresponding equilibrium constants for 36 reactions involving six PAHs and six amino acids. The values for Δ*G*_*R*_ at 25, 100, and 150 °C are listed in Table 4. The Δ*H*_*R*_ and *T*Δ*S*_*R*_ at 25, 100, and 150 °C are listed in Table S1. Looking at Table 5, it is evident that the majority of the 36 investigated reactions are nonspontaneous under the experimental conditions we explored. Reactions can be considered spontaneous when the Δ*G*_*R*_ value is negative. Figure 2 shows an example of two reactions forming glycine or alanine from fluoranthene in a temperature range of 5 to 300 °C. We notice that only the reaction of fluoranthene–alanine is exergonic below ∼39 °C. In general, all 36 PAH–amino acid reactions become more endergonic (less favorable; Table 5) with increasing temperatures. The decrease in favorability of the reaction at increasing temperatures almost directly correlates with the increase of the PAHs’ molecule size, except pyrene. In other words, the more carbon atoms a given PAH contains, the less spontaneous a PAH–amino acid reaction becomes. The precise order in which a PAH–amino acid reaction is more spontaneous is naphthalene (C_10_H_8_) > anthracene (C_14_H_10_) ≈ fluoranthene (C_16_H_10_) > triphenylene (C_18_H_12_) > pyrene (C_16_H_10_) > coronene (C_24_H_12_). A similar trend can be seen for the products. The favorability of the reactions decreases with increasing molecule size of the amino acid except for alanine, which produces favorable reactions in all cases. These trends are depicted in Figure 3, which displays the calculated amino acid concentrations at equilibrium from each PAH. Additionally, we noticed that reactions forming alanine are the only ones that would be above the current experimental detection limit at 25 until 150 °C. The tyrosine-forming reactions produce, by far, the lowest concentrations at equilibrium, with the lowest coronene–tyrosine reaction.

**Table 5:** Indication of the Spontaneity for each PAH–Amino Acid Reaction at 25, 100, and 150 °C^a^

	25 °C			100 °C			150 °C			
Reaction	Δ*H*_*R*_	Δ*S*_*R*_	Δ*G*_*R*_	Δ*H*_*R*_	Δ*S*_*R*_	Δ*G*_*R*_	Δ*H*_*R*_	Δ*S*_*R*_	Δ*G*_*R*_	Spontaneity
NAPH - GLY	-	-	+	-	-	+	-	-	+	nonspontaneous
NAPH - ALA	-	-	-	-	-	+	-	-	+	spontaneous at low T
NAPH - VAL	-	-	+	-	-	+	-	-	+	nonspontaneous
NAPH - LEU	+	-	+	+	-	+	+	-	+	nonspontaneous
NAPH - PHE	+	-	+	+	-	+	+	-	+	nonspontaneous
NAPH - TYR	+	-	+	+	-	+	+	-	+	nonspontaneous
ANTH - GLY	-	-	+	-	-	+	-	-	+	nonspontaneous
ANTH - ALA	-	-	-	-	-	+	-	-	+	spontaneous at low T
ANTH -VAL	+	-	+	-	-	+	-	-	+	nonspontaneous
ANTH - LEU	+	-	+	+	-	+	+	-	+	nonspontaneous
ANTH - PHE	+	-	+	+	-	+	+	-	+	nonspontaneous
ANTH - TYR	+	-	+	+	-	+	+	-	+	nonspontaneous
FLUO - GLY	-	-	+	-	-	+	-	-	+	nonspontaneous
FLUO - ALA	-	-	-	-	-	+	-	-	+	spontaneous at low T
FLUO - VAL	+	-	+	+	-	+	-	-	+	nonspontaneous
FLUO - LEU	+	-	+	+	-	+	+	-	+	nonspontaneous
FLUO - PHE	+	-	+	+	-	+	+	-	+	nonspontaneous
FLUO - TYR	+	-	+	+	-	+	+	-	+	nonspontaneous
PYR - GLY	+	-	+	-	-	+	-	-	+	nonspontaneous
PYR - ALA	-	-	+	-	-	+	-	-	+	nonspontaneous
PYR - VAL	+	-	+	+	-	+	+	-	+	nonspontaneous
PYR - LEU	+	-	+	+	-	+	+	-	+	nonspontaneous
PYR - PHE	+	-	+	+	-	+	+	-	+	nonspontaneous
PYR - TYR	+	-	+	+	-	+	+	-	+	nonspontaneous
TRI - GLY	-	-	+	-	-	+	-	-	+	nonspontaneous
TRI - ALA	-	-	+	-	-	+	-	-	+	nonspontaneous
TRI - VAL	+	-	+	+	-	+	+	-	+	nonspontaneous
TRI - LEU	+	-	+	+	-	+	+	-	+	nonspontaneous
TRI - PHE	+	-	+	+	-	+	+	-	+	nonspontaneous
TRI - TYR	+	-	+	+	-	+	+	-	+	nonspontaneous
CORO - GLY	+	-	+	-	-	+	-	-	+	nonspontaneous
CORO - ALA	-	-	+	-	-	+	-	-	+	nonspontaneous
CORO - VAL	+	-	+	+	-	+	+	-	+	nonspontaneous
CORO - LEU	+	-	+	+	-	+	+	-	+	nonspontaneous
CORO - PHE	+	-	+	+	-	+	+	-	+	nonspontaneous
CORO - TYR	+	-	+	+	-	+	+	-	+	nonspontaneous

a Key: “+” values above and “–“ below 0; Δ*H*_*R*_ = enthalpy of reaction; Δ*S*_*R*_ = entropy of reaction; Δ*G*_*R*_ = Gibbs free energy of reaction; *T* = Temperature. Amino acids: GLY = glycine, ALA = alanine, VAL = valine, LEU = leucine, PHE = phenylalanine, and TYR = tyrosine. PAHs: NAPH = naphthalene, ANTH = anthracene, FLUO = fluoranthene, PYR = pyrene, TRI = triphenylene, and CORO = coronene.

## Discussion

Our experimental results indicate that PAHs do not form amino acids under the investigated conditions (Table 1), and that is consistent with our thermodynamic calculations (Tables 4 and 5). Our theoretic calculations show that a few reactions, mostly involving alanine, are favorable at low temperatures (Table 5). Such reactions would theoretically form a detectable amount of amino acid under our chosen conditions (Figure 3), assuming (i) the reactions had enough time to reach chemical equilibrium and (ii) there were no detrimental factors hindering amino acid formation such as side reactions consuming limiting reactants (e.g., NH_3_) or the presence of insurmountable kinetic barriers for (intermediate) reaction steps. Under our conditions, in order to make a PAH–amino acid reaction favorable, additional energy above that of the amino acid bond formation would be needed. Higher pressure and/or temperature could, in principle, promote some of these reactions due to an increased chance of productive molecular collisions. However, the resulting amino acids would likely hydrolyze quickly after their formation due to such harsh conditions.^51^ Nevertheless, a wide range of amino acids is found in carbonaceous chondrites, which shows that amino acids can survive such conditions.^7,52^ Generally speaking, endergonic reactions and other nonspontaneous physical processes can obtain the required energy for them to occur through a process known as energy coupling, where the endergonic reaction is coupled with an exergonic one, resulting in a globally negative Gibbs free energy. Such energy-coupling phenomena are extremely prevalent in biology (*e.g*., ATP-formation and hydrolysis, electron bifurcation in methanogenesis,^53^ so much so that this constitutes a stepping stone for some prevalent origin-of-life hypotheses (e.g., Sojo et al. (2016)^54^). Nonenzymatic energy-coupling processes have not been described for reactions in asteroids, even though these would be possible in principle. Hence, the energy for the reactions we studied here must be provided by the readiness of the reactants themselves to react under the conditions we explored. Under the assumption that the amino acid formation by PAHs takes place during aqueous alteration, we can imagine that some organic synthesis reactions could be coupled, potentially with the formation of serpentine group minerals (phyllosilicates). We can further imagine that such coupling can only occur when electron transfer between absorbed PAHs on phyllosilicates is possible. That PAHs or other organic compounds are absorbed to minerals and thereby can be altered has been suggested several times^10,17^ and experimentally tested.^13,19,55^ However, mineral-pulled prebiotic chemistry scenarios (*e.g*. Wächtershäuser’s pyrite-pulling; Wächtershäuser (1988)^53^) are backed by scarce experimental evidence. Furthermore, other organic synthesis reactions, like that of methane, could be considered as alternative (to PAHs) sources of organic carbon available for further prebiotically relevant transformations.^56^

Olivine has previously been shown to facilitate the formation of methane and other organic compounds in water.^22−26^ We were not able to analyze the reactant olivine postexperimentally and could not experimentally link a possible olivine alteration with the formation of amino acids either. We can, therefore, only speculate if the alteration of olivine and the resulting formation of phyllosilicates could facilitate amino acid formation. Even if it exists, the time scale of the involved processes could be lengthy and thus hinder the experimental exploration of this reaction landscape. Under Earth conditions, olivine alteration occurs faster with increasing temperatures and is impacted by the aqueous environment.^57^ Carbonaceous chondrites can experience different degrees of aqueous alteration,^30^ and thus may, in principle, provide the suitable temperature range and sufficient fluid water allowing for amino acid formation from PAHs due to phyllosilicate-pulling (via olivine alteration) or due to catalysis by other minerals present in carbonaceous chondrites. In this study, we could not further extend our focus on the influence of olivine alteration over PAH–amino acid reactions, so we leave this question open for future research.

During sample extraction, we observed a yellow-brownish coloration of those sample fluids that contained fluoranthene, ammonium bicarbonate, olivine, and water and were exposed to 150 °C. Such coloration was also observed in an earlier study.^29^ Previously, we assumed that the coloration indicates an alteration of fluoranthene that only occurs above its melting point (∼110 °C; Table 2) and in the presence of olivine. However, it has been found that other PAHs, like pyrene and triphenylene in chloroform, cause a similar coloration after weeks at room temperature and pressure conditions^58^ (photographs included in Figure S1). This observation suggests that a range and combination of factors and such as heat or exposure to light and/or oxygen might trigger or have a catalytic effect on PAHs resulting in the fluid’s coloration. Yet, for instance, merely the addition of heat has shown not to break or promote further structural modification of PAHs in water.^29^ A yellowing/browning of aqueous solutions containing (partially oxidized) hydrocarbons is a common feature in organic chemistry. For instance, the formose reaction—where small aldehydes nonenzymatically condense into larger sugars—experiences a yellowing (due to caramelization) once the formaldehyde fuel has been exhausted.^59^ Carbonyl-containing organics can undergo Maillard reactions and Amadori rearrangements in the presence of amines (e.g., NH_3_), resulting in the browning of the solution. A similar reaction may be at least partially responsible for the observed color change in our samples. We did not probe our samples for the presence of oxidized hydrocarbons, but these species would be the likely primary outcome of PAHs oxidation under aqueous conditions, with oxygen atoms coming either from H_2_O or—if some of the samples were not fully airtight—from atmospheric O_2_. Regardless, the mechanism that causes the coloration does not seem to facilitate amino acid formation (above detectable concentrations), and we recommend further investigation of its origins.

### PAHs as Amino Acids Precursor

Amino acids and PAHs comprise a considerable portion of the soluble material in carbonaceous chondrites.^60^ Some of these amino acids–such as phenylalanine and tyrosine, which were used in this study–contain an aromatic structure. Considering that some amino acids have aromatic moieties, it is appealing to imagine a potential link between PAHs and amino acids. The results from our calculations suggest that there is only a tenuous thermodynamically viable relationship between some of the studied PAHs and amino acids–particularly at low temperatures. However, this was not experimentally corroborated. The absence of experimental evidence for amino acids in our studies could reflect the presence of kinetic barriers resulting in the system not having reached chemical equilibrium in the observed time frame or the reaction(s) progressing too slowly for us to observe product(s).

Over the past few decades, several studies have investigated different ways to form amino acids in asteroids.^10,14,61−64^ For instance, the Strecker synthesis with formaldehyde^14,65^ to form amino acids is (much) more energetically favorable than the PAH–amino acid reactions. If we were to replace a PAH with formaldehyde in our reactions (see Figure 2), all Δ*G*_*R*_ values for the corresponding reactions become negative at 25–150 °C, which would make these reactions indeed thermodynamically favorable (see Table S4).

For the synthesis of amino acids, NH_3_ and a carbon source, like PAHs, are required, which are known to be present in carbonaceous chondrites.^47,66,67^ Our experiments and calculations are representative of our chosen model, and thus we can only extract conclusions limited to such an approach: for instance, the impact of gases onto the PAH–amino acid reactions was not be modeled. Calculations by Shock and Schulte (1990)^10^ suggest that NH_3_, CO_2_, and O_2_ exert an important role the amino acid formation under carbonaceous chondrite conditions. The presence of NH_3_ (or N forming part of organic heterocycles) is essential for amino acid formation and needs no further discussion at this point. We decided to include CO_2_ in this study following Shock and Schulte (1990).^10^ The importance of CO_2_ for the carboxylation of organics in reactions yielding organic acids (or carboxyl-containing organics, such as amino acids) is undeniable,^68^ but it is also possible that such chemical groups can be obtained by (O_2_- or H_2_O-mediated) oxidation of PAHs or PAH fragments. In our thermodynamic calculations, however, we only used CO_2_ if necessary to chemically balance a reaction, and we could therefore not test the influence on the produced concentration as previously suggested. However, if CO_2_ and NH_3_ were available in sufficient quantities, PAHs would easily become the limiting reactant in our studied chemical system, possibly obscuring the role of CO_2_. Our calculations exclude the presence of O_2_, and we took special care to keep our experiments anoxic as explained previously. In practice, the role of O_2_ for amino acid synthesis is debatable given the low O_2_ abundances in carbonaceous chondrites.^30^

The calculations by Shock and Schulte (1990),^10^ reveal that a wider range of amino acids are more favorably formed from pyrene rather than from fluoranthene. Pyrene and fluoranthene share the same molecular formula (C_16_H_10_) but differ in their structure, which is either four fused benzene rings (pyrene) or two benzene rings fused with another one by a 5-carbon ring (fluoranthene). These structural differences (they are constitutional isomers) confer them different properties. The difference in stability most likely results from the diverging structural arrangement of each isomer. But the molecular size also plays a role in the stability, as PAH–amino acid reactions become, in general, thermodynamically less favorable with increasing amounts of carbon atoms (Figure 3), reflecting the trend of increased binding energy per carbon atom. From our analysis, it can be concluded that fluoranthene is the better source for amino acid formation compared to pyrene, and the synthesis of amino acids at higher temperatures, like 100 °C is less favorable, which is in contradiction to the results by Shock and Schulte (1990).^10^ The reasons for the discrepancy between our result and those of Shock and Schulte (1990)^10^ are diverse. The calculations presented here are geared toward the experimental conditions investigated. It focuses on the PAH–NH_4_HCO_3_ equilibrium in an aqueous solution and the formation of amino acids. In contrast, Shock and Schulte (1990)^10^ investigated the equilibrium in a complex mixture considered to be relevant to an asteroidal setting. The adopted CO_2_ and NH_3_ fugacities by Shock and Schulte (1990)^10^ are about 2 orders of magnitude higher (e.g., *f*_NH_3__ = 10^–3^) than pertain to our experimental conditions (e.g., *f*_NH_3__ ≈ 10^–5^). Shock and Schulte tied their high-adopted CO_2_ fugacity to buffering by the mineral assemblages in the meteorite parent body. We note that the results of Shock and Schulte (1990)^10^ also predict that, in contrast to the results reported here, pyrene is more amenable to amino acid formation than fluoranthene. This difference may reflect a difference in the adopted thermodynamic properties, but its origin is difficult to trace back as it is unclear to what data set in Shock and Helgeson (1990)^70^ that Shock and Schulte (1990)^10^ refer to. Additionally, significant differences are Shock and Schulte (1990)^10^ considered a pressure of 100 bar in their calculations and adopted a high oxygen fugacity. Those are two parameters that are not adopted in our set of calculations. A focused theoretical study of the thermodynamic behavior in an asteroidal setting is warranted.

**Figure 4:**
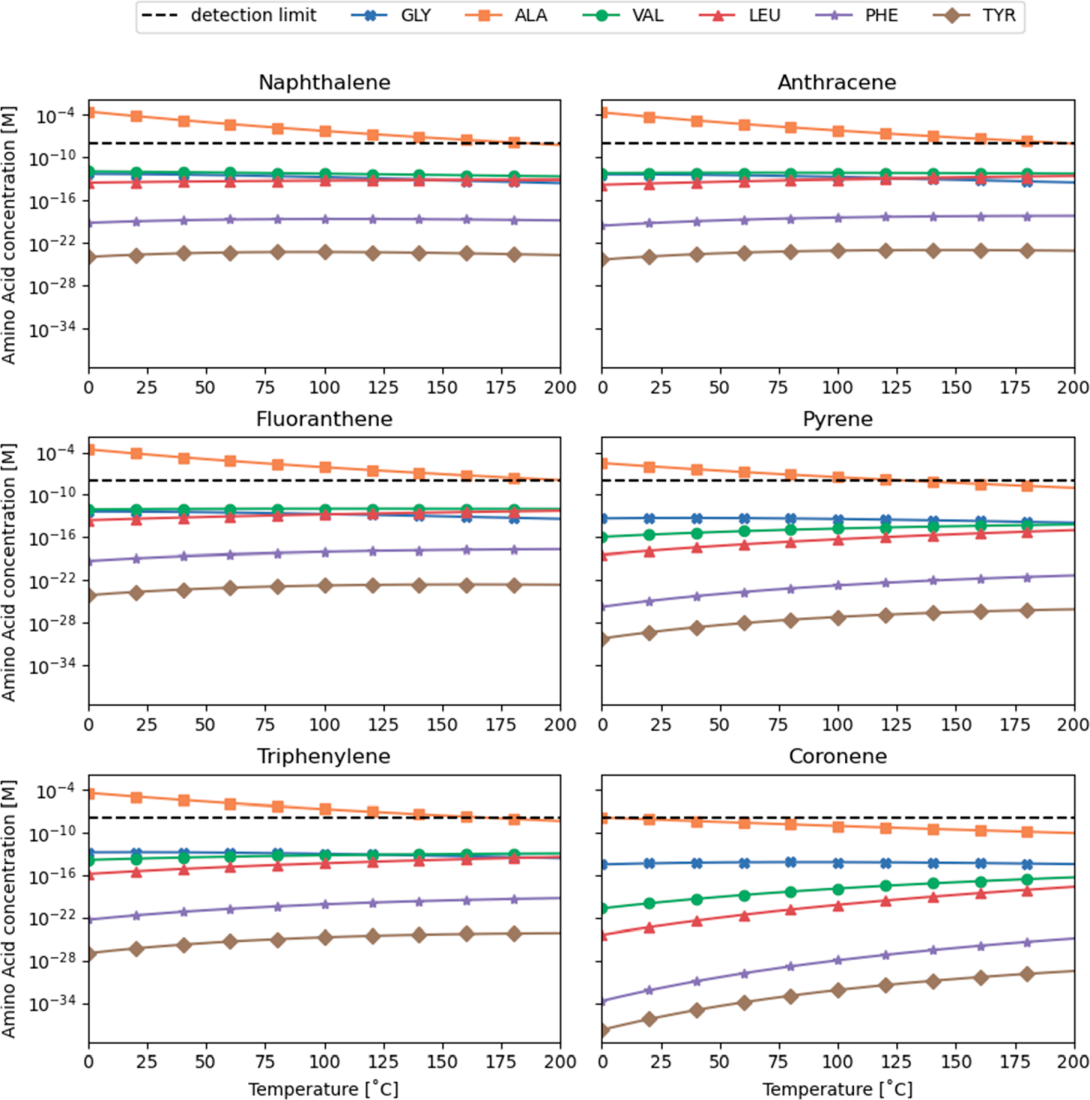
Amino acid concentrations as a function of temperature. The concentrations are calculated with 15 ppm of PAH, 106 ppm of CO_2_, and 19 ppm of NH_3_ as representative values for carbonaceous chondrites.^60^ The indicated detection limit is from the AccQ•Tag method used and serves as a guide as to what would be possible to measure. GLY = glycine, ALA = alanine, VAL = valine, LEU = leucine, PHE = phenylalanine, and TYR = tyrosine.

In our experiments, we only chose temperatures representing the higher end of aqueous alteration temperature in carbonaceous chondrites, whereas with our thermodynamic calculations we also explore temperatures below 100 °C. To evaluate the effect of temperature on the PAH–amino acid reactions under carbonaceous chondrites conditions, we plotted the amino acid concentrations as a function of temperature in Figure 4. For this plot, we used 15 ppm of PAH, 106 ppm for CO_2_, and 19 ppm for NH_3_, which we took as representative for meteorites.^60^ By choosing these values, we simplify the calculations as we compensate for the change of CO_2_ and NH_3_ consumptions as a function of temperature in the reactions. Also, the PAHs become the limiting factor in the PAH–amino acid reactions. Here, it is worth noting that only in the case of naphthalene is a concentration of 15 ppm below its aqueous solubility. As can be seen in Figure 4, each amino acid has a different trend as a function of temperature; alanine is the highest yielding amino acid, and tyrosine is least formed among all reactions. Imposing a pressure as might be expected in carbonaceous chondrites could increase the reaction rate. In our experiments, the pressure was dictated only by the vaporization of water. That resulted in a relative low pressure considering asteroidal conditions, and we could only speculate what an elevated pressure does to the PAH–amino acid reactions. The impact of pressure on the reactions and amino acid yield needs further testing. The impact of temperature, instead, is not only dependent on the type of PAH, but also on factors like the involvement of gases, e.g., CO_2_, in the reaction. Therefore, the observed trends in Figure 4 are, most likely, reflecting the importance of entropy in the different reactions.

Although more experimental and thermodynamic work in the future is required, our thermodynamic calculations imply that PAHs are not particularly good starting materials for amino acid formation.

## Conclusions

In this study, we investigated the potential of PAHs as precursors for amino acid formation in carbonaceous chondrites. We performed laboratory experiments to simulate asteroidal parent body conditions. We used various PAHs in the presence of carbon dioxide and ammonia in aqueous solutions and probed olivine as a catalytic facilitator. Additionally, we performed a theoretical analysis based on the equilibrium thermodynamics of 36 PAH–amino acid reactions. Our experimental results suggest that PAHs are unfavorable sources for forming amino acids since no amino acids were detected. Our thermodynamic calculations further support this empirical observation and indicate that amino acid formation, in particular of alanine, is more favorable at low temperature and remains unfavorable over a wide range of temperatures feasible for aqueous alteration. Other factors such as the impact of pressure on the systems investigated would need to be considered to further test the feasibility of PAHs as precursors for amino acids in carbonaceous chondrites.

## Supporting Information Available

Figure S1, photos of extracted sample material from experiments at 150 °C in comparison to PAH-chloroform solutions; Table S1, the enthalpies Δ*H*_R_ [kJ/mol] and entropies Δ*S*_R_ [kJ/molK] for the formation of 1 mol amino acid from different PAHs in PAH–CO_2_–NH_3_–H_2_O reactions at 25, 100, and 150 °C; Table S2, Gibbs free energy of reaction Δ*G*_*R*_ [kJ/mol] and equilibrium constants *K*_*c*_ for reactions involving the NH_3_–CO_2_–H_2_O-system at 25, 100, and 150 °C; Table S3, concentrations [M] of species in the system and the pH of the full CO_2_–NH_3_–H_2_O system at 25, 100, and 150 °C, calculated with ChemPyl;^50^ and Table S4, Gibbs free energy of reaction values Δ*G*_*R*_ [kJ/mol], equilibrium constants log *K*_*c*_, enthalpies Δ*H*_*R*_ [kJ/mol], and entropies Δ*S*_*R*_ [kJ/molK] for the formation of 1 mol formaldehyde at 25, 100, and 150 °C (PDF)
