# Supplementary material for: Experimental and Theoretical Constraints on Amino Acid Formation from PAHs in Asteroidal Settings
Source: ACS Earth Space Chem. 2022 Feb 15;6(3):468–81. doi: 10.1021/acsearthspacechem.1c00329 (PMC8935471; doi:10.1021/acsearthspacechem.1c00329)
Supplement: Supplementary file 1 — sp1c00329_si_001.pdf [file sp1c00329_si_001.pdf]

Supporting Information

Experimental and Theoretical Constraints on Amino Acid Formation from PAHs in Asteroidal Settings

Claudia-Corina Giese [(1,2)]*, *Inge Loes ten Kate* [(2)], *Martijn P.A. van den Ende* [(3)], *Mariette Wolthers* [(2)], *José C. Aponte* [(4,5,6)], *Eloi Camprubi* [(2)], *Jason P. Dworkin* [(4)], *Jamie E. Elsila* [(4)], *Suzanne Hangx* [(2)], *Helen E. King* [(2)], *Hannah L. Mclain* [(4,5,6)], *Oliver Plümper* [(2)], and *Alexander G.G.M Tielens* [(1)]

[(1)] Leiden Observatory, Faculty of Science, Leiden University, 2300 RA Leiden, The Netherlands

[(2)] Department of Earth Sciences, Faculty of Geosciences, Utrecht University, 3584 CB Utrecht, The Netherlands

[(3)] Université Côte d'Azur, OCA, UMR Lagrange, 06000 Nice, France

[(4)] Solar System Exploration Division, NASA Goddard Space Flight Center, Greenbelt, MD 20771 USA

[(5)] Department of Physics, The Catholic University of America, Washington D. C. 20064, USA

[(6)] Center for Research and Exploration in Space Science and Technology, NASA/GSFC, Greenbelt, MD 20771, USA

*corresponding author: c.c.giese@uu.nl

The following files are available free of charge.

Figure S1 (1 page)

Table S1 (2 pages)

Table S2 (1 page)

Table S3 (1 page)

Table S4 (1page)

Figure S1 displays photos of extracted sample material from experiments at 150 °C in comparison to PAH-chloroform solutions.

Table S1 lists the enthalpies ΔH_R [kJ/mol] and entropies ΔS_R [kJ/molK] for the formation of 1 mol amino acid from different PAHs in PAH-CO_2-NH_3-H_2O reactions at 25, 100, and 150 °C.

Table S2 lists Gibbs free energy of reaction ΔG_R [kJ/mol] and equilibrium constants K_c for reactions involving for the NH_3-CO_2-H_2O-system at 25, 100 and 150 °C.

Table S3 presents the concentrations [M] of species in system and the pH of the full CO_2-NH_3-H_2O-system at 25, 100 and 150 °C, calculated with ChemPy[50].

Table S4 lists the Gibbs free energy of reaction values ΔG_R [kJ/mol], equilibrium constants $log\ K_c$, enthalpies ΔH_R [kJ/mol] and entropies ΔS_R [kJ/molK] for the formation of 1 mol formaldehyde at 25, 100, and 150 °C.

Figure S1. A-D: Extracted sample material from experiments at 150 °C with: **A**: Water, **B**: Fluoranthene, ammonium bicarbonate and olivine powder in water. **C**: Olivine powder in water. **D**: Fluoranthene and ammonium bicarbonate without olivine powder in water. **E-F:** PAH-chloroform solutions at room temperature after two weeks (initially colourless; photograph credit: Nina Kopacz[57]). **E:** Fluoranthene, **F:** Pyrene, and **G:** Triphenylene.

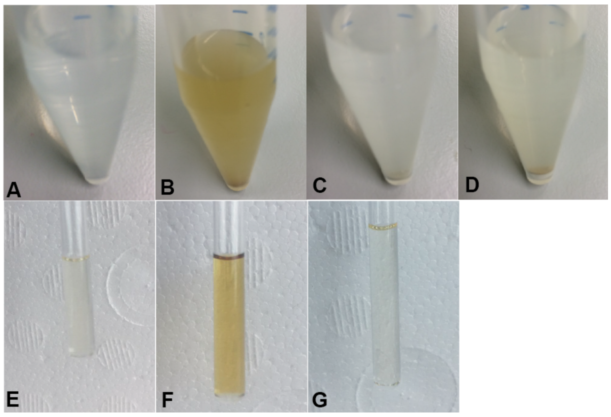

Table S1. Enthalpies ΔH_R [kJ/mol] and entropies ΔS_R [kJ/molK] for the formation of 1 mol amino acid from different PAHs in PAH-CO_2-NH_3-H_2O reactions at 25, 100, and 150 °C. Amino Acids: glycine ($C_2H_5NO_2$), alanine ($C_3H_7NO_2$), valine ($C_5H_{11}NO_2$), leucine ($C_6H_{13}NO_2$), phenylalanine ($C_9H_{11}NO_2$), and tyrosine ($C_9H_{11}NO_3$); PAHs: naphthalene (C_8H_{10}), anthracene ($C_{14}H_{10}$), fluoranthene ($C_{16}H_{10}$), pyrene ($C_{16}H_{10}$), triphenylene ($C_{18}H_{12}$), and coronene ($C_{24}H_{12}$)

	$\Delta H_{R,25}$	$\Delta H_{R,100}$	$\Delta H_{R,150}$	$\Delta S_{R,25}$	$\Delta S_{R,100}$	$\Delta S_{R,150}$
Naphthalene						
$\frac{1}{8} C_{10}H_8 + \frac{3}{4} CO_2 + 1\ NH_3 + \frac{1}{2} H_2O = 1\ C_2H_5NO_2$	-4.64	-17.70	-26.11	-0.15	-0.19	-0.21
$\frac{1}{4} C_{10}H_8 + \frac{1}{2} CO_2 + 1\ NH_3 + 1\ H_2O = 1\ C_3H_7NO_2$	-50.37	-60.78	-67.52	-0.15	-0.18	-0.20
$\frac{1}{2} C_{10}H_8 + 1\ NH_3 + \frac{1}{2} H_2O = 1\ C_5H_{11}NO_2$	-4.73	-9.80	-13.18	-0.15	-0.17	-0.18
$\frac{5}{8} C_{10}H_8 + 1\ NH_3 + 1\frac{5}{2} H_2O = 1\ C_6H_{13}NO_2 + \frac{1}{4} CO_2$	5.54	3.83	2.58	-0.15	-0.15	-0.15
$\frac{5}{6} C_{10}H_8 + \frac{2}{3} CO_2 + 1\ NH_3 + \frac{2}{3} H_2O = 1\ C_9H_{11}NO_2$	14.79	1.49	-7.09	-0.16	-0.20	-0.22
$\frac{19}{24} C_{10}H_8 + 1\frac{1}{12} CO_2 + 1\ NH_3 + \frac{5}{6} H_2O = 1\ C_9H_{11}NO_3$	18.20	-3.45	-17.38	-0.22	-0.28	-0.32
Anthracene						
$\frac{1}{11} C_{14}H_{10} + \frac{8}{11} CO_2 + 1\ NH_3 + \frac{6}{11} H_2O = 1\ C_2H_5NO_2$	-3.21	-15.92	-24.10	-0.15	-0.19	-0.21
$\frac{2}{11} C_{14}H_{10} + \frac{5}{11} CO_2 + 1\ NH_3 + 1\frac{1}{11} H_2O = 1\ C_3H_7NO_2$	-47.12	-56.85	-63.15	-0.15	-0.18	-0.19
$\frac{4}{11} C_{14}H_{10} + 1\ NH_3 + 2\frac{2}{11} H_2O = 1\ C_5H_{11}NO_2 + \frac{1}{11} CO_2$	1.39	-2.29	-4.78	-0.15	-0.16	-0.16
$\frac{5}{11} C_{14}H_{10} + 1\ NH_3 + 2\frac{8}{11} H_2O = 1\ C_6H_{13}NO_2 + \frac{4}{11} CO_2$	14.46	14.47	14.31	-0.14	-0.14	-0.14
$\frac{20}{33} C_{14}H_{10} + \frac{17}{33} CO_2 + 1\ NH_3 + \frac{32}{33} H_2O = 1\ C_9H_{11}NO_2$	25.69	14.71	7.61	-0.15	-0.18	-0.20
$\frac{19}{33} C_{14}H_{10} + \frac{31}{33} CO_2 + 1\ NH_3 + 1\frac{4}{33} H_2O = 1\ C_9H_{11}NO_3$	29.73	10.23	-2.35	-0.21	-0.26	-0.30
Fluoranthene						
$\frac{3}{37} C_{16}H_{10} + \frac{26}{37} CO_2 + 1\ NH_3 + \frac{22}{37} H_2O = 1\ C_2H_5NO_2$	-2.50	-14.94	-22.96	-0.15	-0.19	-0.21
$\frac{6}{37} C_{16}H_{10} + \frac{15}{37} CO_2 + 1\ NH_3 + \frac{4}{37} H_2O = 1\ C_3H_7NO_2$	-46.46	-55.61	-61.55	-0.15	-0.18	-0.19
$\frac{12}{37} C_{16}H_{10} + 1\ NH_3 + 1\frac{14}{37} H_2O = 1\ C_5H_{11}NO_2 + \frac{7}{37} CO_2$	3.51	0.94	-0.86	-0.15	-0.16	-0.16
$\frac{15}{37} C_{16}H_{10} + 1\ NH_3 + 2\frac{36}{37} H_2O = 1\ C_6H_{13}NO_2 + \frac{18}{37} CO_2$	14.51	15.95	16.69	-0.14	-0.13	-0.13
$\frac{20}{37} C_{16}H_{10} + \frac{13}{37} CO_2 + 1\ NH_3 + 1\frac{11}{37} H_2O = 1\ C_9H_{11}NO_2$	27.79	18.66	12.72	-0.15	-0.18	-0.20
$\frac{19}{37} C_{16}H_{10} + \frac{29}{37} CO_2 + 1\ NH_3 + 1\frac{16}{37} H_2O = 1\ C_9H_{11}NO_3$	31.09	13.34	1.87	-0.21	-0.26	-0.29
Pyrene						
$\frac{3}{37} C_{16}H_{10} + \frac{26}{37} CO_2 + 1\ NH_3 + \frac{22}{37} H_2O = 1\ C_2H_5NO_2$	2.73	-9.72	-17.73	-0.15	-0.19	-0.21
$\frac{6}{37} C_{16}H_{10} + \frac{15}{37} CO_2 + 1\ NH_3 + \frac{4}{37} H_2O = 1\ C_3H_7NO_2$	-36.02	-45.17	-51.11	-0.15	-0.18	-0.19
$\frac{12}{37} C_{16}H_{10} + 1\ NH_3 + 1\frac{14}{37} H_2O = 1\ C_5H_{11}NO_2 + \frac{7}{37} CO_2$	24.38	21.82	20.03	-0.15	-0.15	-0.16
$\frac{15}{37} C_{16}H_{10} + 1\ NH_3 + 2\frac{36}{37} H_2O = 1\ C_6H_{13}NO_2 + \frac{18}{37} CO_2$	40.60	42.05	42.79	-0.14	-0.13	-0.13
$\frac{20}{37} C_{16}H_{10} + \frac{13}{37} CO_2 + 1\ NH_3 + 1\frac{11}{37} H_2O = 1\ C_9H_{11}NO_2$	62.63	53.52	47.59	-0.15	-0.18	-0.19
$\frac{19}{37} C_{16}H_{10} + \frac{29}{37} CO_2 + 1\ NH_3 + 1\frac{16}{37} H_2O = 1\ C_9H_{11}NO_3$	64.19	46.46	35.01	-0.20	-0.26	-0.29
Triphenylene						
$\frac{1}{14} C_{18}H_{12} + \frac{5}{7} CO_2 + 1\ NH_3 + \frac{2}{7} H_2O = 1\ C_2H_5NO_2$	-0.25	-12.78	-20.85	-0.15	-0.19	-0.21
$\frac{1}{7} C_{18}H_{12} + \frac{3}{7} CO_2 + 1\ NH_3 + 1\frac{1}{7} H_2O = 1\ C_3H_7NO_2$	-41.77	-51.13	-57.20	-0.15	-0.17	-0.19
$\frac{2}{7} C_{18}H_{12} + 1\ NH_3 + 2\frac{2}{7} H_2O = 1\ C_5H_{11}NO_2 + \frac{1}{7} CO_2$	14.91	11.91	9.84	-0.14	-0.15	-0.16
$\frac{5}{14} C_{18}H_{12} + 1\ NH_3 + 2\frac{6}{7} H_2O = 1\ C_6H_{13}NO_2 + \frac{3}{7} CO_2$	29.35	30.25	30.65	-0.13	-0.13	-0.13
$\frac{10}{21} C_{18}H_{12} + \frac{3}{7} CO_2 + 1\ NH_3 + 1\frac{1}{7} H_2O = 1\ C_9H_{11}NO_2$	44.78	34.99	28.64	-0.15	-0.17	-0.19
$\frac{19}{42} C_{18}H_{12} + \frac{12}{13} CO_2 + 1\ NH_3 + 1\frac{2}{7} H_2O = 1\ C_9H_{11}NO_3$	50.24	31.83	19.94	-0.20	-0.26	-0.29

Table S1. continuation

Coronene

$\frac{1}{18} C_{24}H_{12} + \frac{2}{3} CO_2 + 1\ NH_3 + \frac{2}{3} H_2O = 1\ C_2H_5NO_2$	10.48	-1.52	-9.25	-0.15	-0.18	-0.20
$\frac{1}{9} C_{24}H_{12} + \frac{1}{3} CO_2 + 1\ NH_3 + 1\frac{1}{3} H_2O = 1\ C_3H_7NO_2$	-21.66	-29.89	-35.24	-0.14	-0.17	-0.18
$\frac{2}{9} C_{24}H_{12} + 1\ NH_3 + 2\frac{2}{3} H_2O = 1\ C_5H_{11}NO_2 + \frac{1}{3} CO_2$	55.96	55.18	54.51	-0.14	-0.14	-0.14
$\frac{5}{18} C_{24}H_{12} + 1\ NH_3 + 1\frac{2}{3} H_2O = 1\ C_6H_{13}NO_2 + \frac{2}{3} CO_2$	78.87	82.57	84.74	-0.13	-0.12	-0.11
$\frac{10}{27} C_{24}H_{12} + \frac{1}{3} CO_2 + 1\ NH_3 + \frac{2}{3} H_2O = 1\ C_9H_{11}NO_2$	114.44	108.33	104.30	-0.14	-0.15	-0.16
$\frac{19}{54} C_{24}H_{12} + \frac{15}{27} CO_2 + 1\ NH_3 + 1\frac{24}{27} H_2O = 1\ C_9H_{11}NO_3$	114.48	99.58	89.90	-0.19	-0.24	-0.26

Table S2. Gibbs free energy of reaction ΔG_R [kJ/mol] and equilibrium constants K_c for reactions involving $NH_3(aq, g)$/ $NH^{4+}(aq)$, CO_2 (aq, g)/ $HCO_3^-(aq)$/ $CO_3^{2-}(aq)$, and $H^+(aq)/OH^-(aq)$ at 25, 100, and 150 °C.

	$\Delta G_{R,25}$	$\Delta G_{R,100}$	$\Delta G_{R,150}$	$K_{c,25}$	$K_{c,100}$	$K_{c,150}$
NH_4HCO_3 (s) = CO_2 (aq) + NH_3 (aq) + H_2O (aq)	15.5	-1.3	-15.6	-2.7	0.2	1.9
NH_3 (aq) = NH_3 (g)	-10.6	-4.0	0.6	1.9	0.6	-0.1
NH_3 (aq) + H^+ (aq) = NH^{4+} (aq)	-51.4	-52.1	-53.3	9.0	7.3	6.6
CO_2 (aq) = CO_2 (g)	-8.2	-12.9	-14.0	1.4	1.8	1.7
CO_2 (aq) + OH^- (aq) = HCO_3^- (aq)	-42.8	-39.5	-35.6	7.5	5.5	4.4
HCO_3^- aq + H^+ (aq) = H_2CO_3 (s)	-0.6	2.8	8.0	0.1	-0.4	-1.0
HCO_3^- (aq) = CO_3^{2-} (aq) + H^+ (aq)	59.6	73.9	86.1	-10.4	-10.3	-10.6
H_2O (l) = OH^- (aq) + H^+ (aq)	79.7	86.3	91.4	-14.0	-12.1	-11.3

Table S3. Concentrations [M] of species in system and the pH of the full CO_2-NH_3-H_2O-system at 25, 100, and 150 °C, calculated with ChemPy[50]

	Concentrations in M		
	25°C	100°C	150°C
NH_4HCO_3	$1.2x10^{-3}$	$7.3x10^{-6}$	$1.3\ x10^{-7}$
CO_2 (aq)	$2.9x10^{-4}$	$4.6x10^{-4}$	$6.5x10^{-4}$
CO_2 (g)	$7.9x10^{-3}$	$3.0x10^{-2}$	$3.5x10^{-2}$
CO_3^{2-}	$2.9x10^{-4}$	$2.1x10^{-5}$	$1.4x10^{-6}$
HCO_3^-	$2.8x10^{-2}$	$7.7x10^{-3}$	$2.3x10^{-3}$
H_2CO_3	$3.6x10^{-4}$	$2.0x10^{-4}$	$6.9\ x10^{-5}$
NH_3 (aq)	$8.1x10^{-3}$	$2.4x10^{-2}$	$1.6x10^{-2}$
NH_3 (g)	$1.1x10^{-4}$	$6.4x10^{-3}$	$1.9x10^{-2}$
NH_4^+	$2.9x10^{-2}$	$7.8x10^{-3}$	$2.4x10^{-3}$
H_2O	1.0	1.0	1.0
OH^-	$3.1x10^{-6}$	$5.0x10^{-5}$	$1.4x10^{-4}$
H^+	$3.5\ x10^{-9}$	$1.7x10^{-8}$	$3.9\ x10^{-8}$
pH	8.5	7.8	7.4

Table S4. Gibbs free energy of reaction ΔG_R [kJ/mol], equilibrium constants $log\ K_c$, enthalpies ΔH_R [kJ/mol] and entropies ΔS_R [kJ/molK] for the formation of 1 mol formaldehyde at 25, 100, and 150 °C. Formaldehyde: CH_2O; Amino acids: glycine $C_2H_5NO_2$, alanine $C_3H_7NO_2$, valine $C_5H_{11}NO_2$, leucine $C_6H_{13}NO_2$, phenylalanine $C_9H_{11}NO_2$, and tyrosine $C_9H_{11}NO_3$

	$\boldsymbol{\Delta G_{R,25}}$	$\boldsymbol{\Delta G_{R,100}}$	$\boldsymbol{\Delta G_{R,150}}$	$\boldsymbol{log\ K_{c,25}}$	$\boldsymbol{log\ K_{c,100}}$	$\boldsymbol{log\ K_{c,150}}$	$\boldsymbol{\Delta H_{R,25}}$	$\boldsymbol{\Delta H_{R,100}}$	$\boldsymbol{\Delta H_{R,150}}$	$\boldsymbol{\Delta S_{R,25}}$	$\boldsymbol{\Delta S_{R,100}}$	$\boldsymbol{\Delta S_{R,150}}$
Formaldehyde												
$\frac{3}{2}$ CH_2O + $\frac{1}{2}$ CO_2 + NH_3 = 1 $C_2H_5NO_2$ + $\frac{1}{2}$ H_2O	-206.23	-151.18	-113.54	36.13	21.16	14.02	-420.90	-429.48	-434.91	-0.72	-0.75	-0.76
3 CH_2O + 1 NH_3 = 1 $C_3H_7NO_2$ + 1 H_2O	-292.51	-250.74	-223.68	51.24	35.10	27.61	-461.99	-454.85	-450.21	-0.57	-0.55	-0.54
6 CH_2O + 1 NH_3 = 1 $C_5H_{11}NO_2$+ 1 CO_2 + 2 H_2O	-533.67	-463.28	-419.68	93.49	64.85	51.81	-827.98	-797.94	-778.57	-0.99	-0.90	-0.85
$\frac{15}{2}$ CH_2O + 1 NH_3 = 1 $C_6H_{13}NO_2$+ $\frac{3}{2}$ CO_2 + $\frac{5}{2}$ H_2O	-1338.43	-1170.42	-1067.75	234.48	163.84	131.80	-2047.04	-1962.70	-1908.32	-2.38	-2.12	-1.99
10 CH_2O + 1 NH_3 = 1 $C_9H_{11}NO_2$ + 1 CO_2 + 6 H_2O	-894.97	-784.10	-715.20	156.79	109.76	88.28	-1357.56	-1312.34	-1282.98	-1.55	-1.42	-1.34
19 CH_2O + 2 NH_3 = 1 $C_9H_{11}NO_2$ + 1 CO_2 + 11 H_2O	-1651.70	-1429.54	-1288.95	289.37	200.11	159.11	-2567.57	-2499.83	-2455.72	-3.07	-2.87	-2.76
